# Detection of aquaporin‐4 antibodies for patients with CNS inflammatory demyelinating diseases other than typical MS in Lithuania

**DOI:** 10.1002/brb3.1129

**Published:** 2018-10-03

**Authors:** Eglė Sakalauskaitė‐Juodeikienė, Giedrė Armalienė, Rasa Kizlaitienė, Loreta Bagdonaitė, Nataša Giedraitienė, Dalia Mickevičienė, Daiva Rastenytė, Gintaras Kaubrys, Dalius Jatužis

**Affiliations:** ^1^ Clinic of Neurology and Neurosurgery, Institute of Clinical Medicine, Faculty of Medicine Vilnius University Vilnius Lithuania; ^2^ Department of Physiology, Biochemistry, Microbiology and Laboratory Medicine, Faculty of Medicine Vilnius University Vilnius Lithuania; ^3^ Department of Neurology Hospital of Lithuanian University of Health Sciences Kauno Klinikos Kaunas Lithuania; ^4^ Department of Neurology, Medical Academy Lithuanian University of Health Sciences Kaunas Lithuania

**Keywords:** aquaporin‐4 antibodies, neuromyelitis optica, neuromyelitis optica spectrum disorders

## Abstract

**Objectives:**

Neuromyelitis optica (NMO) is frequently associated with aquaporin‐4 autoantibodies (AQP4‐Ab); however, studies of NMO in Lithuania are lacking. Therefore, the main objective of our study is to assess positivity for AQP4‐Ab in patients presenting with inflammatory demyelinating central nervous system (CNS) diseases other than typical multiple sclerosis (MS) in Lithuania.

**Materials and methods:**

Data were collected from the two largest University hospitals in Lithuania. During the study period, there were 121 newly diagnosed typical MS cases, which were included in the MS registry database. After excluding these typical MS cases, we analyzed the remaining 29 cases of other CNS inflammatory demyelinating diseases, including atypical MS (*n* = 14), acute transverse myelitis, TM (*n* = 8), acute disseminated encephalomyelitis, ADEM (*n* = 3), clinically isolated syndrome, CIS (*n* = 2), atypical optic neuritis, ON (*n* = 1), and NMO (*n* = 1). We assessed positivity for AQP4‐Ab for the 29 patients and evaluated clinical, laboratory, and instrumental differences between AQP4‐Ab seropositive and AQP4‐Ab seronegative patient groups.

**Results:**

AQP4‐Ab test was positive for three (10.3%) patients in our study, with initial diagnoses of atypical MS (*n* = 2) and ADEM (*n* = 1). One study patient was AQP4‐Ab negative despite being previously clinically diagnosed with NMO. There were no significant clinical, laboratory, or instrumental differences between the groups of AQP4‐Ab positive (3 [10.3%]) and negative (26 [89.7%]) patients.

**Conclusions:**

AQP4‐Ab test was positive for one‐tenth of patients with CNS inflammatory demyelinating diseases other than typical MS in our study. AQP4‐Ab testing is highly recommended for patients presenting with not only TM and ON but also an atypical course of MS and ADEM.

## 1 INTRODUCTION

Neuromyelitis optica (NMO)—autoimmune inflammatory central nervous system (CNS) disease characterized by severe attacks of optic neuritis (ON) and longitudinally extensive transverse myelitis (LETM; Wingerchuk et al., 2015). A report by Antoine Portal (1742–1832), the first physician to king Louis XVIII, represents probably the first account of visual loss in a patient with spinal cord inflammation but no brain pathology in the Western literature (Jarius & Wildemann, 2012). French term *neuro‐myélite optique aiguë* was first used by Eugène Devic (1858–1930) in a paper communicated on the occasion of the *Congrès Français de Médecine* in Lyon in 1894, where he denoted a novel syndrome characterized by acute myelitis and optic neuritis (Jarius & Wildemann, 2013).

In recent years, NMO has raised enormous interest among scientists and clinical neurologists, fueled by the detection of a highly specific serum immunoglobulin G autoantibody targeting the astrocytic water channel aquaporin‐4 (AQP4) by Dr. Lennon and colleagues in 2004 (Jarius & Wildemann, 2013; Lennon et al., 2004; Lennon, Kryzer, Pittock, Verkman, & Hinson, 2005). This discovery has made clear that in most cases NMO is not a subform of multiple sclerosis (MS), but an autoimmune condition with an immunopathogenesis distinct from that of MS despite considerable overlap in clinical presentation and paraclinical findings (Jarius, Wildemann, & Paul, 2014). In 2007, the term NMO spectrum disorders (NMOSD) was introduced to include AQP4‐Ab seropositive patients with limited or inaugural forms of NMO: first‐attack LETM, recurrent or bilateral ON (Wingerchuk, Lennon, Lucchinetti, Pittock, & Weinshenker, 2007). The term also encompasses the cerebral, diencephalic, and brainstem lesions that occur in a minority of patients with otherwise typical NMO (Wingerchuk et al., 2015).

Population‐based studies from Europe, South‐East and Southern Asia, the Caribbean, and Cuba suggest that the incidence and prevalence of NMO ranges from 0.05–0.4 and 0.52–4.4 per 100,000, respectively (Pandit et al., 2015). Typical age at NMO onset peaks at approximately 35–45 years, but NMO may also manifest in children and the elderly (Huppke et al., 2010; Jarius et al., 2014). Female preponderance is substantially higher in seropositive (9–10:1) than in seronegative patients (2:1; Wingerchuk, 2009). The majority of NMO cases are sporadic, although rare familial cases have also been reported (Matiello et al., 2010). It is known that about 70%–80% of NMO cases are associated with aquaporin‐4 autoantibodies (AQP4‐Ab; Jarius, Franciotta, et al., 2010). The detection of AQP4‐Ab is essential as it justifies consideration of long‐term immunosuppression (Kimbrough et al., 2012; Sellner et al., 2010; Trebst et al., 2014), as interferon‐beta (IFN‐beta), natalizumab and fingolimod have been reported to be inefficacious or even harmful when used for the NMO treatment (Kowarik, Soltys, & Bennett, 2014).

The percentage of NMO in Asia and the West Indies was known to be almost 50% of CNS demyelinating disorders (Kowarik et al., 2014). NMO was considered to be a rare disorder in Caucasians; however, this view was based on few studies with small patient populations from tertiary hospitals (Wu, Zhang, & Carroll, 2008).

The prevalence of AQP4‐Ab positive patients is unknown in Lithuania. Therefore, the main objective of our study is to assess positivity for AQP4‐Ab in patients presenting with demyelinating inflammatory CNS diseases other than typical MS in Lithuania (an atypical course of MS; acute transverse myelitis [TM]; severe, atypical ON; NMO; acute disseminated encephalomyelitis [ADEM]; and clinically isolated syndrome [CIS]). The secondary objectives of this study are to evaluate clinical, laboratory, and instrumental differences between AQP4‐Ab seropositive and AQP4‐Ab seronegative patient groups. To our knowledge, this is the first study to assess the frequency of AQP4‐Ab in patients with demyelinating CNS diseases other than typical MS in Lithuania.

## 2 MATERIALS AND METHODS

### 2.1 Participants

Patients were selected from the Departments of Neurology, MS centers of the two largest university hospitals in Lithuania (2,944,459 inhabitant population, 2014): (Statistics of Lithuania) Vilnius University Hospital Santaros klinikos and Hospital of Lithuanian University of Health Sciences Kauno klinikos. According to the Lithuanian MS registry database, there were 121 newly diagnosed typical MS cases in Lithuanian MS centers during the study period. Considering growing NMO recognition in Caucasian populations, we performed a countrywide, cross‐sectional exploratory study from November 2013 to January 2015.

Twenty‐nine patients over 18 years of age who presented with demyelinating CNS diseases other than typical MS were included in the study. We assessed positivity for AQP4‐Ab in patients presenting with an atypical course of MS that did not fulfill the 2010 McDonald MRI Criteria for lesion dissemination in time and space (Polman et al., 2011), acute TM, severe, atypical ON, NMO, ADEM, and clinically isolated syndrome (CIS). We evaluated clinical, laboratory, and instrumental differences between AQP4‐Ab seropositive and AQP4‐Ab negative patient groups.

NMO diagnosis was made according to the revised diagnostic criteria of Wingerchuk, Lennon, Pittock, Lucchinetti, and Weinshenker (2006) After this study, clinical diagnoses were revised and some patients were diagnosed with NMO or NMOSD (Table 1) based on the International Consensus Diagnostic Criteria for neuromyelitis optica spectrum disorders (Wingerchuk et al., 2015). The CIS group included cases with a single inflammatory demyelinating episode except NMOSD. The atypical MS course group included patients who did not strictly fulfill the 2010 McDonald MRI Criteria for lesion dissemination in time and space (there were no lesions in periventricular or juxtacortical areas and demyelinated lesions were small in shape or localized only in the brainstem and spinal cord); however, all these patients had at least two severe relapses and fulfilled other 2010 McDonald criteria (Polman et al., 2011). Diagnoses of ADEM, TM, and ON were based on previously known diagnostic criteria (Bermel & Balcer, 2013; Krupp, Banwell, & Tenembaum, 2007; Transverse Myelitis Consortium Working Group, 2002). The exclusion criteria were as follows: a history of chronic disease of the immune system other than the demyelinating CNS diseases mentioned above, any concomitant diseases causing neurological physical disability, psychiatric disorders, diseases affecting cognitive functions, or a history or presence of malignancy and active systemic infection.

**Table 1:** Case reports for AQP4‐Ab positive patients

1. A 38‐year‐old woman with no antecedent illness or vaccination	
2013 January–May	Presented with diplopia, moderately severe paraparesis of legs, urine retention, progressive truncal and bilateral lower extremity numbness (Th10 sensory level). Cervical and thoracic spine MRI: T2 hyperintense LETM lesion with gadolinium enhancement at the C5‐TH11 level. Brain MRI: T2 hyperintense nonenhancing lesions in the periependymal surfaces of the fourth ventricle, brainstem, and cerebellum. ADEM diagnosed. Treated with high‐dose methylprednisolone, then plasma exchange. Oral prednisolone introduced. Urosepsis was treated with antibiotics. EDSS 7.0
2013 June	Remission. Oral Prednisolone continued, Azathioprine introduced. EDSS 6.5
2013 November	Remission. Test for AQP4‐Ab positive. Revised clinical diagnosis, AQP4‐Ab seropositive NMOSD diagnosed. Azathioprine continued. EDSS 6.0
2014 May	Remission. Thoracic spine MRI: T2 hyperintense nonenhancing lesion at Th4. Brain MRI: T2 hyperintense nonenhancing periaqueductal lesion. Azathioprine continued. EDSS 4.0
2015 January	Remission. Azathioprine continued. EDSS 3.5
2. A 37‐year‐old woman with a previous history of trigeminal neuralgia	
2011 May	Presented with acute retrobulbar ON, treated with peroral methylprednisolone in regional hospital. EDSS 1.0
2011 September	Relapsed with left hemiparesis. Brain MRI: small, nonenhancing periventricular and brainstem lesions not fulfilling the 2010 McDonald MRI Criteria for lesion dissemination in time and space. Oligoclonal bands in CSF. Atypical MS diagnosed and patient treated with high‐dose methylprednisolone. IFN‐beta introduced. EDSS 2.5
2012 July	Relapsed with left hemiparesis, urine retention, and trigeminal neuralgia, treated with high‐dose methylprednisolone. IFN‐beta continued. EDSS 3.5
2013 February	Brain MRI: no T2 hyperintense lesions.
2013 August	Relapsed with paresis of right leg, left hemiparesis, and urine retention, treated with high‐dose methylprednisolone. IFN‐beta continued. EDSS 4.0
2014 April	Relapsed with paraparesis of legs, paresis of left arm, truncal and bilateral lower extremity numbness (Th4 sensory level). Cervical and thoracic spine MRI: T2 hyperintense nonenhancing lesions at the C5‐Th1, Th3‐4, and Th7‐8 levels. Treated with high‐dose methylprednisolone. IFN‐beta discontinued, azathioprine introduced. EDSS 6.5
2014 December	Remission. Brain MRI: T2 hyperintense nonenhancing lesions, not fulfilling the 2010 McDonald MRI Criteria for lesion dissemination in time and space. Test for AQP4‐Ab positive. Revised clinical diagnosis, NMO diagnosed. Azathioprine intolerance. Oral Prednisolone introduced, Rituximab considered. EDSS 5.0
3. A 42‐year‐old woman, shortly after influenza	
2003 August	Presented with paraparesis of legs, urine retention, truncal, and bilateral lower extremity numbness (Th4 sensory level). Cervical and thoracic spine MRI: T2 hyperintense LETM lesion from C2 to *conus medullaris*, spinal cord swelling. Treated with high‐dose methylprednisolone. Oral Prednisolone introduced. EDSS 3.5
2004 November	Remission. Brain MRI: no T2 hyperintense lesions
2005 May	Relapsed with paraparesis of legs. Atypical MS diagnosed and treated with plasma exchange. EDSS 3.0
2006 September	Relapsed with acute retrobulbar ON, treated with high‐dose methylprednisolone. EDSS 3.0
2007 March	Remission. Brain MRI: no T2 hyperintense lesions. Glatiramer acetate prescribed
2008 April	Relapsed with severe paraparesis of legs, treated with high‐dose methylprednisolone. EDSS 4.5
2008 June	Relapsed with paraparesis of legs. Oligoclonal bands in CSF. Treated with high‐dose methylprednisolone. Glatiramer acetate continued. EDSS 3.5
2010 April	Relapsed with paraparesis of legs, urine retention, treated with high‐dose methylprednisolone. Brain MRI: no T2 hyperintense lesions. Glatiramer acetate continued. EDSS 3.5
2010 December	Relapsed with right leg paresis, treated with high‐dose methylprednisolone. Glatiramer acetate continued. EDSS 4.0
2011 May	Relapsed with severe right leg paresis, severe ataxia. Cervical and thoracic spine MRI: T2 hyperintense nonenhancing lesions around central cord at Th2‐Th5, C2‐C4 levels, spinal cord atrophy at Th2‐Th5. Treated with high‐dose methylprednisolone, then plasma exchange. EDSS 6.5.
2011 July	Relapsed with paraparesis of legs, ataxia, imperative voiding. Mitoxantrone introduced. EDSS 6.5
2011 July–2012 November	Remission. Mitoxantrone infusions. EDSS decreased from 6.5 to 4.0
2013 June	Remission. IFN‐beta introduced. EDSS 4.0
2013 October	Relapsed with paraparesis of legs, ataxia, truncal, and bilateral lower extremity numbness (Th4 sensory level), imperative voiding. Treated with plasma exchange. IFN‐beta continued. EDSS 6.5
2014 February	Test for AQP4‐Ab positive. Clinical diagnosis revised, NMO diagnosed. IFN‐beta discontinued, azathioprine introduced. EDSS 5.5
2014 September	Remission. Azathioprine continued. EDSS 5.0

Note

ADEM, acute disseminated encephalomyelitis; AQP4‐Ab, aquaporin‐4 autoantibodies; CSF, cerebrospinal fluid; EDSS, expanded disability status scale; IFN‐beta, interferon‐beta; LETM, longitudinally extensive transverse myelitis; MRI, magnetic resonance imaging; MS, multiple sclerosis; NMO, neuromyelitis optica; NMOSD—neuromyelitis optica spectrum disorders; ON, optic neuritis.

Neurologic and ophthalmologic examinations were performed. For each patient, a questionnaire consisting of an anamnesis and socio‐demographic data was completed by the examiner. Magnetic resonance imaging (MRI), visual and somatosensory evoked potentials (EPs), and cerebrospinal fluid (CSF) analyses were either collected retrospectively or performed during admission. Clinical (duration of the disease, EDSS, and neurological examination findings), instrumental (total number of spinal cord MRI lesions, number of spinal cord MRI lesions extending ≥3 vertebral segments (VS), number of brain MRI lesions, abnormal visual or somatosensory EPs, and optic disk atrophy), and laboratory (oligoclonal bands in CSF) data were evaluated. Differences between AQP4‐Ab positive and AQP4‐Ab negative patient groups were compared. The time between the onset of clinical myelitis symptoms and spinal cord MRI for the patients was 30 days or less. All 29 patients were tested for AQP4‐Ab during remission, when the AQP4‐Ab test became available in Lithuania (from November 2013).

The study was approved by the Lithuanian Bioethics Committee on January 27, 2011 (No. L‐12‐01/2), and all patients provided written informed consent.

### 2.2 AQP4‐antibody assay

For the detection of AQP4‐Ab, an indirect immunofluorescence test with aquaporin‐4‐transfected and non‐transfected cells (EU90) was used (Euroimmun, Germany). Substrates were incubated with a 1:10 diluted patient sample; if the reaction was positive, specific human IgG antibodies against aquaporin‐4 reacted with the transfected cells of substrate. The attached antibodies were stained with fluorescein‐labeled anti‐human antibodies and made visible with a fluorescence microscope. The samples dilution starting point was 1:10*.* Samples were stored up to 10 days at temperatures between +2°C and +8°C.

### 2.3 Statistical analyses

Continuous variables were expressed as mean ± *SD* and/or median (range), checked for normal distribution using the Shapiro–Wilk test and compared by Mann–Whitney *U* test for non‐normally distributed variables. Fisher's exact test was used to compare categorical variables between groups. Due to the exploratory nature of the study, no adjustment for multiple comparisons was made. A *p*‐value <0.05 was considered statistically significant. The data were processed using Microsoft Office Excel 2007 and R Studio, version 0.99.879.

## 3 RESULTS

### 3.1 Characteristics of study population

**Figure 1:**
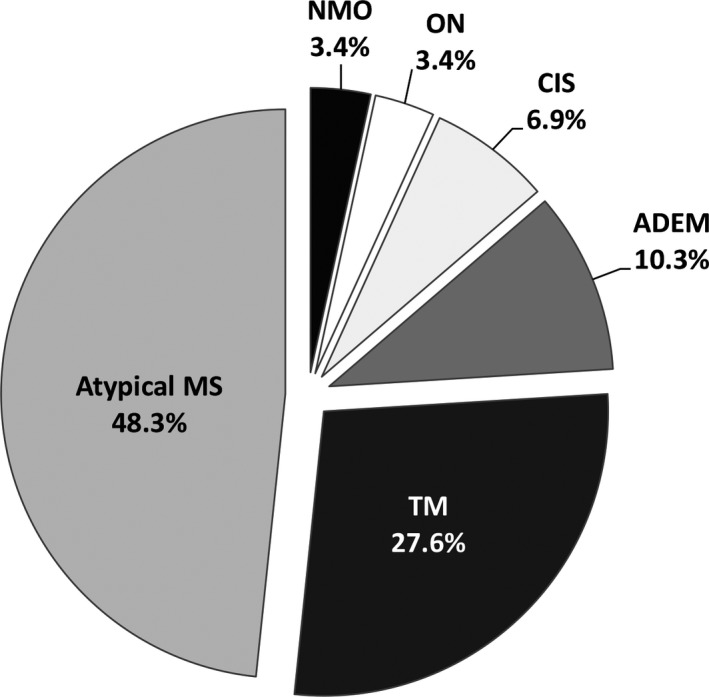
Diagnoses of all study patients at study entry (*n* = 29). ADEM, acute disseminated encephalomyelitis; CIS, clinically isolated syndrome; MS, atypical multiple sclerosis; NMO, neuromyelitis optica; ON, optic neuritis; TM, transverse myelitis

Twenty‐nine patients were included in the study: seven (24.1%) males and 22 (75.9%) females 18 years of age or older (mean age was 41.3 ± 12.5 years, from 22 to 64 years). At study entry, the largest proportions of patients were diagnosed with atypical MS—14 (48.3%) and TM—8 (27.6%; Figure 1). One patient's initial diagnosis was NMO (the diagnosis was based on Wingerchuk‘s clinical 2006 criteria: two absolute criteria were positive and two out of three supportive criteria were positive; serum testing for AQP4‐Ab was not available before this study).

**Figure 2:**
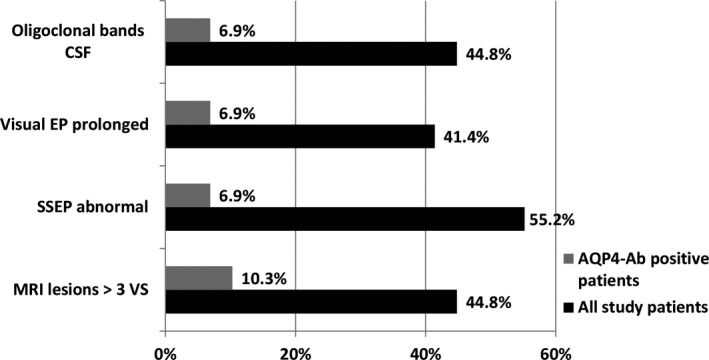
Laboratory and instrumental data for AQP4‐Ab positive patients (*n* = 3) and all study patients (*n* = 29). AQP4‐Ab, aquaporin‐4 autoantibodies; CSF, cerebrospinal fluid; EP, evoked potentials; MRI, magnetic resonance imaging; SSEP, somatosensory evoked potentials; VS, vertebral segments

Oligoclonal bands in CSF were found in 13 (44.8%) patients, visual EPs were abnormal for 12 (41.4%), somatosensory EPs—for 16 (55.2%) patients, 13 (44.8%) patients had ≥1 spinal cord MRI lesions extending ≥3 VS (Figure 2). At study entry, 11 (37.9%) patients received symptomatic medication, six (20.7%) received disease‐modifying therapies (DMTs), six (20.7%) received immunosuppressants, and six (20.7%) were under observation. All patients receiving DMTs were initially diagnosed with an atypical MS.

The AQP4‐Ab test was positive for three (10.3%) patients with initial diagnoses of atypical course MS (two patients) and ADEM (one patient). While the samples dilution starting point was 1:10, for the three AQP4‐Ab positive samples, fluorescence at titer 1:100 was considered a strong positive. One study patient was AQP4‐Ab negative despite being previously clinically diagnosed with NMO. For short case reports of the three AQP4‐Ab positive patients, see Table 1.

### 3.2 Comparison between AQP4‐Ab seropositive and AQP4‐Ab negative patient groups

The duration of the disease in the AQP4‐Ab seropositive and AQP4‐Ab seronegative group was 55.0 ± 57.9 months and 48.6 ± 61.3 months, respectively, when the AQP4‐Ab testing was performed. There were no significant clinical, laboratory, or instrumental differences between the groups of AQP4‐Ab positive (3 [10.3%]) and negative (26 [89.7%]) patients (Table 2).

**Table 2:** Characteristics of AQP4‐Ab positive and AQP4‐Ab negative patient groups

Clinical, instrumental, and laboratory data	Patient groups	
	AQP4−Ab (+), *n* = 3	AQP4−Ab (−), *n* = 26
Duration of the disease (months)	55.0 ± 57.9	48.6 ± 61.3
EDSS	6.0 ± 0.0	4.4 ± 1.7
Number of spinal cord MRI lesions extending ≥3 VS	1.0 ± 0.0; 1 (1–1)	0.6 ± 0.8; 0 (0–2)
Number of spinal cord MRI lesions	2.0 ± 1.7; 1 (1–4)	2.7 ± 1.9; 2 (0–8)
Number of brain MRI lesions	1.3 ± 1.2; 2 (0–2)	3.9 ± 4.4; 3 (0–14)
Oligoclonal bands in CSF	2 (66.7%)	11 (42.3%)
Abnormal visual EPs	2 (66.7%)	11 (42.3%)
Abnormal somatosensory EPs	2 (66.7%)	14 (53.9%)
Optic disk atrophy	1 (33.3%)	7 (26.9%)

Note

AQP4‐Ab, aquaporin‐4 autoantibodies; CSF, cerebrospinal fluid; EDSS, expanded disability status scale; EPs, evoked potentials; MRI, magnetic resonance imaging; VS, vertebral segments; *p* > 0.05

## 4 DISCUSSION

In North America, Australia, and Europe NMO patients represent a small fraction (1%–2%) of Caucasians with inflammatory white matter diseases (Kowarik et al., 2014). As about 70%–80% of NMO cases are associated with aquaporin‐4 antibodies, AQP4‐Ab testing is an essential tool for NMO diagnosis and consideration of treatment options, especially long‐term immunosuppression (Sellner et al., 2010). It was also suggested that NMO spectrum should be broadened to include AQP4‐Ab positive patients with monophasic or more limited phenotypes (Sato et al., 2013).

AQP4‐Ab test was positive for three (10.3%) patients with demyelinating CNS diseases other than typical MS in our study. In a previous study, Korean patients with inflammatory demyelinating CNS diseases (including MS patients) were tested for AQP4‐Ab, and 106 (out of 388) were found to be positive (Kim, Kim, Li, Jung, & Kim, 2012). In contrast, it has been reported that NMO is rare among ON patients in the population of southern Finland; of the 300 patients with suspected ON, only three patients (1.6%) were found to be positive for AQP4‐Ab (Siuko et al., 2014). Further, AQP4‐Ab is relatively rare among patients with acute monosymptomatic ON: AQP4‐Ab were detected in only eight (5.8%) out of 139 patients from European countries and Turkey who presented with acute monosymptomatic ON (Jarius, Frederikson, et al., 2010). In this same study, all the 32 MS patients were tested negative for AQP4‐Ab (Jarius, Frederikson, et al., 2010). We strongly believe that the implementation of AQP4‐Ab testing could greatly improve NMO and NMOSD diagnosis in Lithuania.

One NMO patient in the present study was AQP4‐Ab seronegative. About 12% to 30% of patients with NMO or NMOSD remain AQP4‐Ab negative (Marignier et al., 2013). Some of our AQP4‐Ab negative patients may be positive for antibodies to myelin oligodendrocyte glycoprotein (anti‐MOG). It was previously reported that antibodies to MOG were detected in some AQP4‐Ab negative patients manifesting clinical and neuroimaging signs of NMO or NMOSD (Zamvil & Slavin, 2015). Furthermore, the results of one observational study showed that serum peptide reactivities may also have the potential to distinguish between both NMOSD subgroups and MS (Metz et al., 2016).

Oligoclonal bands (OCB) were detected in the CSF for the two AQP‐4 positive patients in our study. Two studies in which the OCB were analyzed, but OCB were not the primary goal, showed that OCB were detected in CSF for the 531 (83.2%) of typical MS patients in Vilnius (unpublished data) and for the 88 (73.3%) typical MS patients in Kaunas MS centers (Balnytė, 2012), Lithuania. Sometimes OCB are found as a mirror pattern, reflecting the OCB pattern in the blood, but then another systemic inflammatory diseases (including autoimmune disorders, other than MS, paraneoplastic diseases, and infections of the CNS) could be detected (Thompson, 2005).

All patients receiving DMTs were initially diagnosed with atypical MS in the present study. Two AQP4‐Ab positive patients, who were initially diagnosed with atypical MS, were also on DMTs (one received glatiramer acetate, other—IFN‐beta); however, their disability progressed. The diagnoses were revised after the AQP4‐Ab testing, these patients were switched to azathioprine, and the course of the disease stabilized. Therefore the present study illustrates the importance of AQP4‐Ab testing. The detection of AQP4‐Ab is substantial, because it justifies consideration of long‐term immunosuppression, while DMTs have been reported to be inefficacious or even harmful when used for the NMO treatment (Kowarik et al., 2014).

The time between the onset of clinical myelitis symptoms and spinal cord MRI for patients in the present study was 30 days or less (for the first myelitis episodes); however, we did not perform spinal cord MRI for our patients during all repeated spinal cord attacks. The timing of MRI in the evolution of NMOSD may influence the length of the imaged lesion: early imaging may miss a long lesion, and late imaging may reveal discontinuous or short lesions or no lesions; therefore, it is suggested that short spinal cord lesion does not exclude the diagnosis of NMOSD (Flanagan et al., 2015). However, almost half (44.8%) of the patients in our study had ≥1 spinal cord MRI lesions extending ≥3 VS. Even though LETM was reported to be the most specific radiological finding supporting NMO diagnosis in adult patients (Wingerchuk et al., 2006), short TM is not uncommon in NMOSD and does not exclude NMOSD diagnosis (Flanagan et al., 2015) and decision to perform AQP4‐Ab testing. Brain MRI has also an increasingly important role in the differential diagnosis of NMO and NMOSD, particularly from MS, as differentiating these conditions is of prime importance because early initiation of immunosuppressive therapy is the key to preventing attack‐related disability in NMOSD (Kowarik et al., 2014), as shown in the case reports in our study.

We acknowledge the limitations of the study: small sample size and possible referral bias as our hospitals are tertiary referral centers. We hope to address these issues in the future studies by using larger sample sizes and including more patients from regional hospitals.

In conclusion, AQP4‐Ab test was positive for one‐tenth of patients presenting with inflammatory demyelinating CNS diseases other than typical MS in our study. There were no significant clinical, laboratory, or instrumental differences between the groups of AQP4‐Ab positive and negative patients. AQP4‐Ab testing is highly recommended for patients presenting with not only TM and ON but also an atypical course of MS and ADEM.

## CONFLICT OF INTERESTS

The authors state no conflict of interests.
